# Acute effects of strength training interventions on subjective, neuromuscular, and biochemical fatigue parameters in elite youth soccer players

**DOI:** 10.3389/fspor.2026.1742295

**Published:** 2026-03-04

**Authors:** Björn Kadlubowski, Michael Keiner, Klaus Wirth, Robert Csapo

**Affiliations:** 1 Centre for Sport Science and University Sports, Department of Sport and Human Movement Science, University of Vienna, Vienna, Austria; 2 Vienna Doctoral School of Pharmaceutical, Nutritional and Sport Sciences, University of Vienna, Vienna, Austria; 3 Department of Training and Exercise Science, German University of Health and Sport, Berlin, Germany; 4 Department of Sport Science, University of Applied Sciences Wiener Neustadt, Wiener Neustadt, Austria

**Keywords:** creatine kinase, monitoring, performance, plyometric, recovery

## Abstract

This study examined acute neuromuscular, biochemical, and subjective fatigue responses to two strength training protocols—back squats with calf raises vs. back squats with plyometric exercises—in elite youth soccer players. The aim was to track the progression and resolution of fatigue over a 72 h recovery period and evaluate the practical feasibility of both protocols within high-performance training schedules. Thirty-two male athletes from a national youth elite training center participated in a crossover-controlled study. Each player completed both interventions, separated by an 8-day washout period. A comprehensive test battery assessed neuromuscular performance via the countermovement jump (CMJ), drop jump, isometric mid-thigh pull, adductor squeeze, and sit-and-reach test; biochemical markers via creatine kinase (CK); and subjective markers using the Hooper Index, visual analog scale (VAS), and session rating of perceived exertion (sRPE). Measurements were taken at baseline and 24, 48, and 72 h post-exercise. No significant interaction effects (training modality × time) were found (*p* > 0.05), indicating comparable fatigue and recovery trajectories for both protocols, whereas significant main effects of time were observed across all parameters, including CMJ [*F*(3,26) = 29.373, *p* < 0.001, *η*^2^ = 0.772] and CK [*F*(3,26) = 51.504, *p* < 0.001, *η*^2^ = 0.856]. Fatigue peaked between 24 and 48 h post-exercise and returned to baseline by 72 h. Subjective fatigue (Hooper, VAS) mirrored objective markers. Both traditional and reactive strength training induced short-term fatigue that resolved within 72 h, supporting their safe implementation in elite youth training programs, provided adequate recovery is allowed between sessions.

## Introduction

Strength and explosiveness are fundamental physical qualities in high-performance soccer players, underpinning key actions such as sprinting, jumping, tackling, and rapid changes of direction that collectively contribute to overall match performance (1–4). Consequently, structured strength and plyometric training interventions are widely implemented within elite youth development programs to enhance neuromuscular performance and prepare players for the increasing physical demands of professional soccer (5–7).

Recent findings by Crotty et al. (8), and Pandy et al. (9), highlight the crucial role of the plantar flexors during acceleration. Traditional strength training, typically involving squats and calf raise exercises, is primarily performed with controlled movement velocities and moderate-to-high external loads and aims to increase maximal force production and foundational strength capacities (10). These adaptations are considered essential for supporting sprint acceleration, jump performance, and force transmission during high-intensity soccer actions. In contrast, plyometric or reactive strength training emphasizes rapid eccentric–concentric muscle actions and focuses on enhancing stretch–shortening cycle efficiency, reactive strength, and neuromuscular coordination (11, 12). Kadlubowski et al. (13), investigated the effects of a 6-month training intervention combining back squats either with standing calf raises or with reactive strength training. Their results showed that both interventions may improve sprint, jump and maximal strength performance, with no significant differences between them. Based on these findings, both approaches could be considered viable components of youth elite soccer training programs.

However, when evaluating the suitability of a training method, it is not sufficient to consider performance improvements alone. The physiological strain imposed on players, and the resulting fatigue, plays a decisive role in determining how well strength and conditioning measures can be integrated into the broader context of soccer-specific development, particularly technical and tactical skill acquisition, which remains the top priority in youth training. Moreover, excessive strain may interfere with match performance and recovery during competitive seasons, underscoring the need for carefully balanced training programs. Given the distinct biomechanical and neuromuscular demands associated with traditional and reactive strength training, it is plausible that different fatigue trajectories may emerge. Understanding these differences is essential for optimizing training loads, supporting athlete development, and ensuring that strength and conditioning measures enhance rather than disrupt overall soccer performance. In applied terms, identifying the short-term fatigue and recovery characteristics of each modality can help coaches schedule strength and plyometric sessions appropriately within weekly training microcycles.

Fatigue responses to strength training are task-dependent and reflect both central and peripheral mechanisms, which vary with the nature of the activity (14–16). Fatigue is a multidimensional phenomenon encompassing both subjective perceptions of effort or tiredness and objective, measurable declines in performance capacity. Kluger et al. (17), distinguish between perceptual fatigue—the subjective feeling of exhaustion—and performance fatigability—the actual decline in physical performance. In the context of strength and conditioning, these dimensions interact dynamically, as neuromuscular impairments can influence perceptual strain, while heightened perceived effort may, in turn, affect neuromuscular performance. Evaluating both subjective and neuromuscular markers therefore provides a more comprehensive understanding of post-exercise fatigue in elite youth soccer players. Traditional strength training, typically involving high external loads and controlled movement velocities, initiates fatigue primarily through central (neural) mechanisms, such as reduced motor unit recruitment. As the session progresses, peripheral factors, such as disruptions in excitation, contraction coupling, accumulation of metabolic by-products, and increased mechanical stress, may further contribute to the observed decline in performance (14, 18, 19). However, the extent and nature of fatigue are strongly influenced by the specific loading scheme (number of sets and repetitions, relative intensity, and recovery duration) which determine the balance between neural and peripheral contributions. For instance, eccentric-biased prescriptions typically induce greater muscle damage and slower recovery than other contraction modes, even at similar external loads (20, 21). At matched intensities, sets performed closer to momentary failure tend to elicit larger acute neuromuscular and metabolic disturbances and slower recovery within 24–48 h (21, 22). As training progresses, peripheral factors such as impaired excitation–contraction coupling, accumulation of metabolic by-products, and mechanical microtrauma further contribute to performance decrements (16, 23). In contrast, reactive strength training (RST), which emphasizes rapid SSC actions, elicits transient neuromuscular fatigue without substantial metabolic disturbance or alterations in tendon stiffness (24, 25). These differences are likely to result in distinct recovery trajectories, which in turn affect how easily each training modality can be integrated into a soccer training schedule.

Empirical studies have shown that neuromuscular performance and biochemical markers such as creatine kinase may remain impaired for 24–72 h after soccer match play and after resistance or plyometric exercise (22, 26–29). In addition to such objective markers, sRPE has become a widely used tool to capture athletes’ global perception of internal load, as values obtained within a short period following exercise are considered to provide a valid representation of the overall session demand while minimizing recall bias (30, 31). However, most available data come from adult players and from studies examining single exercise modalities or single fatigue dimensions, leaving limited evidence on how different strength-training approaches compare when multiple fatigue markers are assessed concurrently in elite youth athletes.

This study aimed to compare the fatigue responses elicited by two commonly used strength training modalities—traditional strength training (squats combined with calf raises) and a program emphasizing reactive strength training (squats combined with plyometric exercises)—over a 72 h recovery period in youth soccer players. To ensure a comprehensive understanding of training-induced fatigue, a multi-faceted methodological approach was chosen, incorporating subjective, biochemical, and neuromuscular measures. While the acute neuromuscular and biochemical responses to strength training are influenced by variables such as training volume and time under tension, hypertrophy-oriented resistance-based exercise stimuli typically impose greater mechanical strain and sustained neural activation than the reactive, plyometric stimulus. We therefore hypothesized that it would result in more pronounced and prolonged fatigue-related responses compared to the combined strength-plyometric session.

## Materials and methods

This study employed a crossover-controlled design to examine acute neuromuscular, biochemical, and subjective fatigue responses to two strength training protocols in elite youth soccer players. This classification of players followed internationally recognized criteria (32, 33). Participants were recruited from the under−17 (U17) and under-19 (U19) teams of a German elite youth academy, representing the highest competitive level below professional soccer and competing in the highest national junior division (U17/U19 Junioren-Bundesliga) for these age categories.

The two training protocols included (i) traditional strength training, consisting of back squats combined with standing calf raises (CR-BS), and (ii) a multi-modal exercise protocol emphasizing reactive strength training that consisted of back squats combined with plyometric exercises (PLY-BS). All players completed the CR-BS protocol prior to the PLY-BS protocol, separated by an 8-day washout phase. The order was determined to ensure methodological consistency and because traditional loading was considered a more controlled protocol before exposure to higher reactive demands.

**Figure 1:**
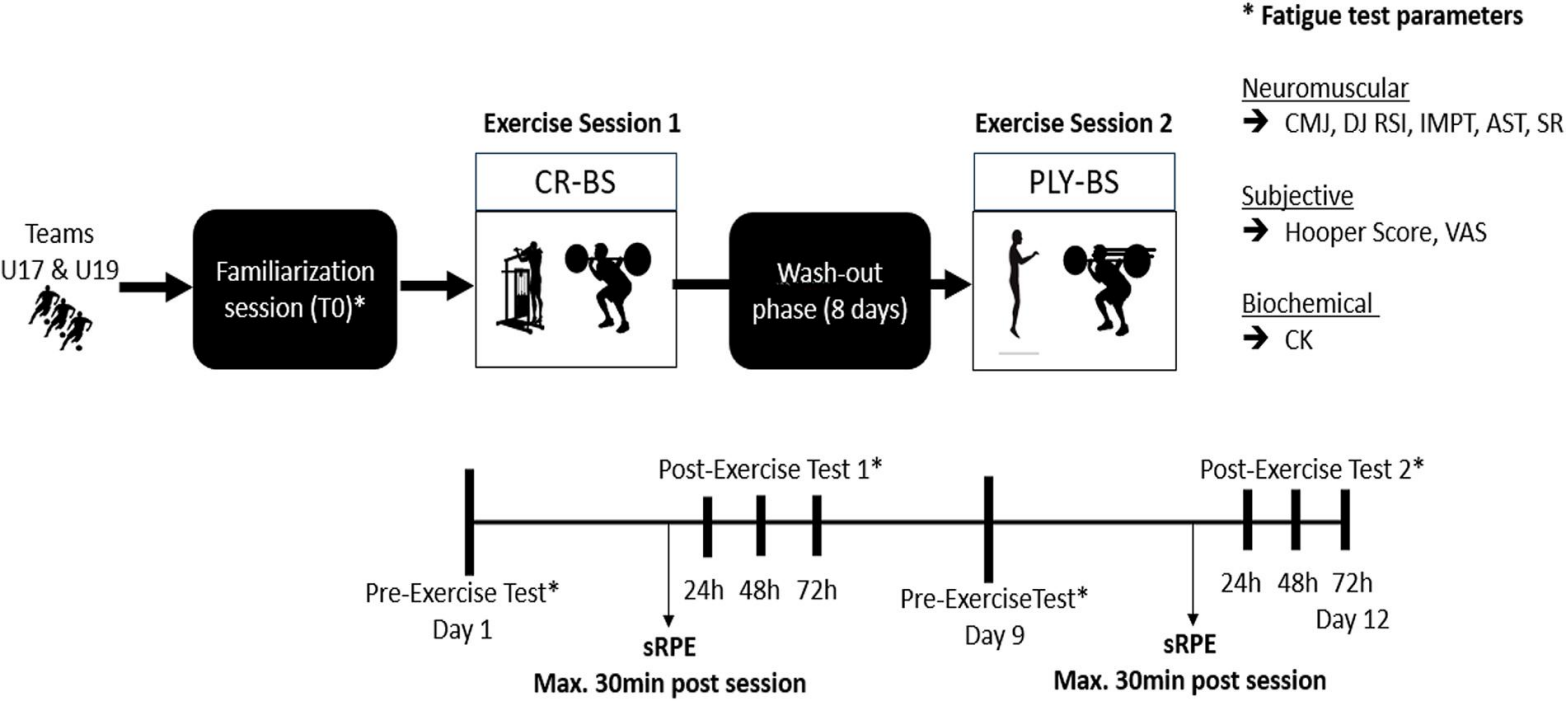
Experimental design. U19, under-19; U17, under-17; PLY-BS, black squat and plyometric training group; CR-BS, black squat and standing calf raise group; CMJ, countermovement jump; DJ RSI, drop jump reactive strength index; IMPT, isometric mid-thigh pull; AST, adductor squeeze test; SR, sit and reach test; VAS, visual analogue scale; CK, creatine kinase; sRPE, session rating of perceived exertion.

Fatigue assessments were conducted at four time points: pre-exercise (baseline), and 24, 48, and 72 h post-exercise. sRPE was recorded within a maximum of 30 min after each exercise session. While several tests have been validated specifically in soccer populations, others have been primarily validated in athletic or resistance-training contexts. These measures were included based on their established physiological relevance, high reliability, and frequent application in applied sports science research to assess neuromuscular performance and fatigue-related responses. A visual overview of the study design is provided in Figure 1.

### Subjects

Thirty-two male youth soccer players (age: 16.5 ± 0.7 years; height: 1.78 ± 0.06 m; mass: 72.2 ± 7.3 kg) from the U17 and U19 squads of a German elite youth academy participated in this study. All players had been playing soccer since early childhood, with an average training age of approximately 12 years. All participating players typically engaged in 4–5 weekly soccer-specific training sessions (90 min each), focused on technical and tactical development, along with weekend competitions. In addition, they regularly engaged in two weekly athletic training sessions. These sessions included sprinting, jumping, stabilization exercises, and basic strength work; however, none of the players had prior experience with systematic strength training programs.

The study-specific exercise sessions were conducted over a two-week period during the Easter break—a phase without league matches. In the two weeks prior to the study, no strength or reactive strength training was performed, and in the two days preceding baseline testing no soccer was played and no athletic training was conducted. These measures ensured that players entered the intervention in a standardized and rested condition. To further minimize residual fatigue, the intervention and pre-assessment sessions were scheduled eight days apart.

Due to logistical constraints –players were living at home and attending different schools—systematic monitoring of sleep and nutritional behavior was not feasible. However, all participants were reminded to ensure adequate sleep and maintain a balanced diet. Given the athletes’ competitive level and professional aspirations, it can reasonably be assumed that they adhered to generally healthy lifestyle habits.

None of the subjects reported injuries at the time of testing. All participants—and, in the case of minors, their legal guardians—were informed about the experimental risks associated with the study before providing written informed consent. Ethical approval was obtained from the institutional review board of the German University of Health and Sport (DHGS-EK-2024-008) and the study was conducted in accordance with the Helsinki Declaration.

### Measures and procedures

To comprehensively monitor neuromuscular performance, flexibility, and fatigue status, a structured and validated test battery was administered under standardized conditions. All testing sessions were conducted in the morning hours (09:00–11:00) in the gym under standardized indoor conditions to minimize circadian variation and environmental influences. Players refrained from strenuous training for at least 48 h prior to baseline testing. A familiarization session was conducted in a rested state one week prior to the first exercise session. During this session, players performed three trials off the following tests to assess parameters indicative of neuromuscular fatigue: sit and reach test (SR); isometric adductor squeeze test (AST); countermovement jump (CMJ); drop jump (DJ); and isometric mid-thigh-pull (IMTP)). All other measures were obtained in a single trial. A one-minute passive recovery period was provided between individual tests. This interval was selected to reduce the risk of cumulative fatigue while preserving neuromuscular readiness and has been shown to be sufficient for reliable performance assessment in similar neuromuscular testing protocols (34, 35). The consistent use of this rest interval across all testing sessions ensured methodological standardization.

On the two exercise days, the players performed only one trial per test immediately before and within a maximum of 30 min after the exercise session. The test sequence was selected based on current scientific evidence (36) and best-practice recommendations to ensure validity and minimize intra-session fatigue and test interference (37). The consistent use of this rest interval across all testing sessions ensured methodological standardization. Testing lasted approximately 15 min.

Physical testing was supervised by four experienced coaches to ensure strict adherence to protocols and maximize measurement reliability. To maintain consistency and minimize inter-rater variability, all assessments were conducted by the same individuals throughout the study. One coach was responsible for collecting creatine kinase samples, another administered the subjective questionnaires, and two coaches oversaw neuromuscular fatigue assessments. A one-minute break was provided between all tests.

Subjective fatigue markers were assessed first to provide insight into the athletes’ recovery status prior to physical exertion. Flexibility was then measured using the SR, a reliable and widely accepted method for evaluating lower back and hamstring flexibility (Cuenca-Garcia et al., 2022). This was followed by the AST, CMJ, and DJ, all of which served as indicators of neuromuscular fatigue particularly relevant to soccer performance. To avoid potentiation or fatigue effects influencing subsequent tests, the IMTP was performed last to assess maximal muscle strength, as the contractile history of prior maximal or near-maximal efforts may acutely enhance subsequent explosive performance through post-activation potentiation (Tillin & Bishop, 2009; Blazevich & Babault, 2019). Finally, blood samples were collected for creatine kinase analysis.

Jump performance was assessed using a dual force plate system (FD4000, VALD Performance, Brisbane, Australia) sampling at 1,000 Hz, with jump height calculated using the impulse-momentum method in accordance with validated protocols (38). During the baseline test, a one-minute rest was provided between individual trials and a 15 min rest between different jump types.

For the CMJ, players were instructed to bend their knees to 90°, then perform an explosive vertical jump with hands fixed on the hips to ensure a stable posture (39). DJs were performed from a height of 30 cm. Participants stood with their hands on their hips and stepped horizontally off the box, aiming to minimize ground contact time and maximize jump height. To reduce contact duration, they were instructed not to let their heels touch the ground upon landing. The RSI, calculated as the ratio of jump height (cm) and ground contact time (s), was used as gross indicator of DJ performance. Both CMJ height and DJ RSI have demonstrated good-to-excellent reliability in soccer players and are considered sensitive markers of neuromuscular status (40–43).

Flexibility of the hamstrings and lower back was assessed using the SR with a Sit and reach box (Baseline, PhysioSupplies, Groningen, Niederlande). Participants sat on the floor with both legs extended and the soles of their feet flat against the front panel of the box. With one hand placed on top of the other and arms fully extended at shoulder height, they were instructed to take a deep breath, exhale, and reach forward as far as possible without bending their knees. If a trial was deemed invalid due to knee flexion or other form violations, an additional attempt was permitted. The SR is widely used in athletic populations to assess flexibility and can also serve as indirect indicator of muscular fatigue. Reductions in flexibility may reflect transient increases in muscle stiffness and reduced range of motion resulting from exercise-induced muscle damage, inflammation, and elevated passive tension (20, 44). Although the sit-and-reach test has been most extensively validated in adult populations, it has also been widely used in youth and adolescent athletes as a practical field-based measure of posterior chain flexibility. However, flexibility assessments in youth athletes may be influenced by growth- and maturation-related factors, including changes in limb length and musculoskeletal development, which should be considered when interpreting absolute values (45). Previous studies have demonstrated high test–retest reliability in adult males (ICC > 0.97) (46), making it a practical tool for both performance and recovery monitoring.

The AST was used to quantify isometric hip adduction strength and assess fatigue-related strength loss. The test was performed in a supine position with the hips at 45° flexion, the knees at 90°, and the arms positioned alongside the body. A specific dynamometer (KangaTech KT360, North Melbourne, VIC, Australia) was placed between the knees, and participants performed a maximal squeeze for 5 s. Research in semi-professional soccer players has shown high test-retest reliability (ICC > 0.80) (47).

Maximal isometric force and rate of force development (RFD) were assessed using the IMTP. Participants stood on a force platform (FD4000, VALD Performance) in a standardized partial squat position, with the grip fixed at mid-thigh level. A metal bar with a rough surface was connected to the floor via an adjustable chain, which was individually adjusted to each participant's anthropometrics to ensure consistent positioning and tension. Participants performed a maximal pull lasting 5 s, and peak force (N) was calculated. The IMTP is considered a reliable and sensitive measure of neuromuscular fatigue and maximal strength capacity, with ICC values reported in male youth soccer players ranging from 0.84 to 0.98 (48).

The Hooper questionnaire was administered to assess the athletes’ perceived response to the preceding exercise session (49). Each player rated four subjective parameters: (i) sleep quality during the previous night, (ii) perceived stress, (iii) fatigue, and (iv) muscle soreness. Each variable was evaluated using a 7-point Likert scale ranging from 1 (‘very, very low’ or ‘very good’) to 7 (‘very, very high’ or ‘very poor’). The overall Hooper score (HS) was calculated as the sum of these four individual ratings, yielding a single composite measure of the athletes’ psychophysiological status, with higher total scores indicating greater perceived fatigue and poorer recovery status, whereas lower scores reflect a more recovered state. The Hooper Index has been shown to be a practical and sensitive tool for tracking match-induced fatigue and overall wellness in team-sport athletes, with small typical error relative to the smallest worthwhile change and good signal-to-noise characteristics when monitoring recovery in professional soccer players (50). Additionally, its subcomponents, including fatigue, stress, sleep quality, and muscle soreness, have been linked to training load variations in elite soccer, supporting its validity as a psychophysiological indicator of athlete status in both individual and team sport contexts (51).

sRPE was used as a subjective measure of internal load and collected exclusively after completion of the exercise sessions. This tool captures the athletes’ perceived exertion and is commonly used to complement objective indicators such as heart rate or external training load (30, 52). Within a maximum of 30 min after each exercise session, players were asked to rate their overall perceived effort during the session on a 10-point modified Borg scale, ranging from 0 (“rest”) to 10 (“maximal exertion”) (30). sRPE is widely used as a valid indicator of internal training load, showing strong associations with physiological markers such as heart rate and blood lactate across a range of team-sport populations (53–55). It also demonstrates good reliability when collected within a short time window after exercise and provides a practical means to quantify global effort in both training and competition settings (56, 57).

Subjective perceptions of mental and muscular fatigue were evaluated using the VAS, a widely accepted tool for assessing symptoms such as pain, fatigue, and soreness due to its simplicity, sensitivity, and established validity and reliability (58, 59). The VAS consists of a 10 cm horizontal line with descriptive anchors at each end: “no muscle soreness” on the left and “worst imaginable muscle soreness” on the right. To standardize the evaluation, participants performed a barefoot bodyweight squat to a 90° knee angle before indicating the intensity of soreness on the VAS. They were instructed to mark their perceived level along the line.

To evaluate exercise-induced muscle damage, creatine kinase (CK)—one of the most commonly used biomarkers for detecting muscle tissue disruption following physical exertion—was analyzed (60, 61). CK has consistently been shown to rise in response to strenuous exercise and match play in team-sport athletes, supporting its validity as an indicator of muscle disruption (62, 63). Although absolute responses may vary substantially between individuals, repeated measurements demonstrate acceptable within-subject consistency when pre-analytical variation is controlled (64). Capillary blood samples were collected from the fingertip and analyzed using the SimplexTAS™ 101 analyzer with a parameter-specific reagent cartridge (Hitado GmbH, Dreihausen, Möhnesee, Germany). Quality-control procedures were performed in accordance with the manufacturer's guidelines, including internal system checks prior to each testing session. Parameter-specific, single-use reagent cartridges containing pre-calibrated reagents were used for all analyses, minimizing operator-dependent variability.

### Exercise interventions

To minimize confounding effects of prior fatigue, overall training load was deliberately reduced during the study period. The two experimental sessions (PLY-BS and CR-BS), each lasting up to 60 min, were performed once per week, with no additional athletic or soccer training scheduled on the respective days. The first session was conducted on a Monday, followed by the second on the Tuesday of the following week.

Between the experimental sessions, only three moderate-intensity training sessions were conducted—on the Thursday, Friday and Saturday following the first session. These sessions explicitly excluded sprinting, jumping, and strength-based exercises to avoid inducing neuromuscular fatigue. This approach provided a controlled training environment and minimized carryover effects between the two experimental conditions. A detailed weekly overview of the experimental and concurrent training sessions is provided as Supplementary Material.

The exercise stimuli implemented in the two experimental conditions consisted of the BS exercise with an Olympic barbell and standing CR on a calf raise machine (CR-BS), or BS combined with plyometric training (PLY-BS). Prior to the first experimental session, participants completed a familiarization session to ensure proper execution. Both protocols followed a hypertrophy-oriented intensity format. Training loads were based on those used during a strength training session conducted three weeks earlier, which also involved the same barbell and calf raise machine. The intensity for both BS and CR exercises was prescribed according to a repetition scheme using moderate loads (from 8 to 12 repetitions per set at 60% to 80% of 1-RM) (65). Importantly, all sets were performed to volitional fatigue, in line with the hypertrophy-focused approach described by Kadlubowski et al. (13). The same weight was used in both experimental sessions.

Warm-up routines were performed both with the barbell and on the calf raise machine, consisting of two unloaded sets of 20 repetitions and one set of 10 repetitions with an additional 20 kg load. In preparation for the plyometric exercises, participants completed ten minutes of coordinative drills and dynamic stretching. Warm-up sets were clearly separated from the working sets and not included in the total working set count.

The CR-BS session began with the BS exercise, followed by the CR. Initial weight selection aimed to allow all players to complete the prescribed repetitions with proper form, avoiding compensatory movements. If execution was incorrect, the set was terminated and the load reduced by 5 kg in the following set. Conversely, if more than 12 repetitions to volitional fatigue were possible, the load was increased by 5 kg in the subsequent set. Further details of the CR-BS exercise session are provided in Table 1.

**Table 1:** Exercise parameters for the back squat and standing calf raise exercise (CR-BS) session.

Sets BS/CR	Repetitions BS/CR	Weight (kg) BS (Mean ± SD)/CR (Mean ± SD)	Breaks after sets (min) BS/CR
5/5	8–12/8–12	(80 ± 10)/(50 ± 15)	3/3

BS, back squat; CR, standing calf raise at machine; SD, standard deviation; m, meter; kg, kilogram; min., minutes.

The exercise stimulus for the PLY-BS condition included reactive bounding jumps over mini hurdles, which required a controlled slow eccentric phase and emphasized horizontal force development. The spacing of the hurdles and the box height were adjusted in accordance with the hypertrophy block described by Kadlubowski et al. (13). The protocol also included drop jumps from a 45 cm height, emphasizing a brief eccentric phase, short ground contact times (<250 ms), and explosive vertical force development. Players, who were already familiar with various forms of plyometric exercise, were specifically instructed to minimize knee and hip flexion upon landing (66). Following completion of the plyometric component, participants performed the BS exercise as described above. A detailed overview of the plyometric exercise protocol combined with the BS is presented in Table 2.

**Table 2:** Exercise parameters for the back squat and plyometric exercise (PLY-BS) session.

Exercises	Sets/repetitions	Weight (kg) BS (Mean ± /SD)	Breaks after repetitions (s)	Breaks after sets (min.)
Hurdle jumps (30 cm) −1 m distance between hurdles	4/6	–	30	2
Drop jumps (45 cm)	4/6	–	20	2
BS	5/8–12	80 ± 10	–	3

BS, back squat; SD, standard deviation; cm, centimeter; m, meter; kg, kilogram; min., minutes.

### Statistical analysis

The significance level for all statistical tests was set at *p* < 0.05. The normality of data was assessed using the Kolmogorov–Smirnov test, and results are expressed as mean ± SD for all parameters across training modalities and time points. For the neuromuscular variables, intraclass correlation coefficients (ICC) and 95% confidence intervals (95%) were calculated as measures of test-retest reliability.

To assess the assumption of sphericity, which is a prerequisite for repeated-measures ANOVA, Mauchly's test of sphericity was applied to all within-subject factors. In cases where this assumption was violated (*p* < 0.05), Greenhouse-Geisser corrections were used to adjust the degrees of freedom and preserve statistical validity.

To examine changes over time and differences in response to the two training protocols (PLY-BS vs. CR-BS), a two-way repeated-measures ANOVA (2 × 4) was performed for each outcome measure, considering both training modality (PLY-BS, CR-BS) and time (PRE-EX, Post24, Post48, Post72) as within-subjects factors. Where significant interaction or main effects were identified, Bonferroni-adjusted pairwise comparisons were conducted to locate differences between specific time points.

For the ANOVA results the effect sizes for the global effects were calculated via the partial square of eta (*η*^2^). In general, effect sizes of *η*^2^ ≥ 0.25 are classified as large, *η*^2^ ≥ 0.1 as moderate and *η*^2^ ≥ 0.01 as small (67).

## Results

Descriptive statistics including means, standard deviations, intraclass correlation coefficients (ICC), and 95% confidence intervals for all baseline performance measures are reported in Table 3. All outcome variables were normally distributed, confirming the suitability of parametric statistical analyses.

**Table 3:** Reliability statistics and 95% confidence intervals of the neuromuscular performance parameter baseline tests.

Fitness test	Mean ± SD	ICC (95% CI)
Counter-movement jump	40.29 ± 3.89	0.991 (0.98–0.99)
Isometric mid-tigh pull	2,418.30 ± 255.74	0.848 (0.72–0.92)
Drop-jump	1.63 ± 0.39	0.974 (0.95–0.99)
Sit-and-reach	9.23 ± 3.06	0.994 (0.99–1.00)
Adductor strength test	38.98 ± 9.21	0.980 (0.96–0.99)

SD, standard deviations; 95% CI, confidence intervals.

**Figure 2:**
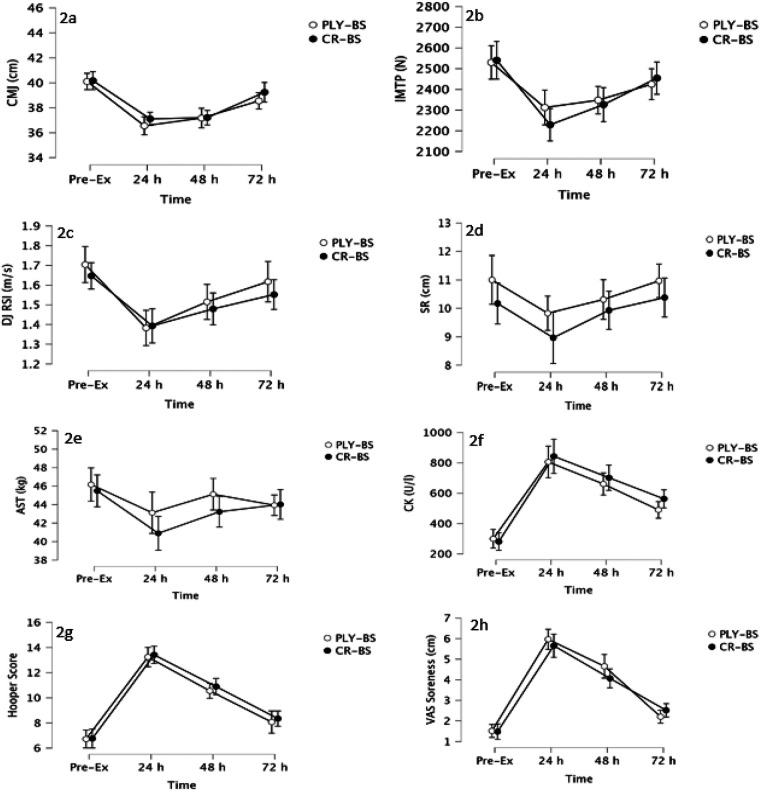
**(a–h)** time course of the various fatigue test parameters at baseline and 24, 48, and 72 h post-exercise. **(a)** Countermovement jump performance; **(b)** Isometric mid-thigh-pull pertformance; **(c)** Drop jump reactive strength index; **(d)** Sit and reach test; **(e)** Adductor squeeze test; **(f)** Creatine kinase levels; **(g)** Hooper questionnaire scores; **(h)** Visual analogue scale results. PLY-BS, back squat and plyometric training group; CR-BS, back squat and standing calf raise on a machine group; Pre-Ex, baseline test; h, hours; cm, centimeter; N, force; m/s, meter per second; kg, kilogram; CK, Creatine kinase; U/L, units per liter; VAS, visual analogue scale; IMPT, isometric mid-thigh pull; Drop jump reactive strength index; SR, sit and reach test; AST, adductor squeeze test; VAS, visual analogue scale; cm, centimeter.

The results of the repeated-measures ANOVAs used to analyze the effects of training modality (PLY-BS, CR-BS) and *time* (PRE-EX, Post24, Post48, Post72) on the performance parameters are presented in the following sections. The time course of all outcome measures by training modality is illustrated in Figures 2a–h. No significant time × training modality interactions were found for any of the outcome measures, indicating that the two training protocols elicited comparable temporal patterns of change. Likewise, main effects of training modality were non-significant across all variables, whereas significant main effects of time were observed for each outcome, reflecting systematic changes over the post-exercise recovery period.

For the CMJ (Figure 2a), no significant interaction [F(3,26) = 1.465, *p* = 0.247, *η*p^2^ = 0.149] or main effect of training modality [F(1,28) = 0.984, *p* = 0.330, *η*p^2^ = 0.034] was found. However, a significant main effect of time was observed [F(3,26) = 29.373, *p* < 0.001, *η*p^2^ = 0.772], indicating performance changes across the post-exercise time points.

The IMTP (Figure 2b) showed no significant interaction effect [F(3,26) = 1.005, *p* = 0.406, *η*p^2^ = 0.104] or main effect of training modality [F(1,28) = 0.156, *p* = 0.696, *η*p^2^ = 0.006], while a significant main effect of time [F(3,26) = 15.061, *p* < 0.001, *η*p^2^ = 0.635] was found.

DJ (Figure 2c) performance also showed no significant interaction effect [F(3,26) = 0.032, *p* = 0.406, *η*p^2^ = 0.031] and no significant differences between training modalities [F(1,28) = 0.612, *p* = 0.441, *η*p^2^ = 0.021], but a significant main effect of time was observed [F(3,26) = 17.200, *p* < 0.001, *η*p^2^ = 0.665].

For SR (Figure 2d), no statistically significant interaction [F(3,26) = 0.058, *p* = 0.663, *η*p^2^ = 0.058] or main effect of training modality [F(1,28) = 1.670, *p* = 0.207, *η*p^2^ = 0.056] was detected, however, a moderate main effect of time was observed [F(3,26) = 6.868, *p* = 0.002, *η*p^2^ = 0.435].

The AST (Figure 2e) showed neither a significant interaction [F(3,26) = 0.176, *p* = 0.164, *η*p^2^ = 0.176] nor a significant main effect of training modality [F(1,28) = 1.055, *p* = 0.313, *η*p^2^ = 0.036], while a significant change over time [F(3,26) = 10.261, *p* < .001, *η*p^2^ = 0.542] was found.

CK (Figure 2f) levels showed neither a statistically significant interaction [F(3,26) = 0.121, *p* = 0.332, *η*p^2^ = 0.121] nor a main effect of training modality [F(1,28) = 0.534, *p* = 0.471, *η*p^2^ = 0.019], but they increased significantly over time [F(3,26) = 51.504, *p* < 0.001, *η*p^2^ = 0.856], indicating elevated muscle damage markers post-exercise.

For the Hooper score (Figure 2g) no significant time × training modality interaction [F (3,26) = 0.145 *p* = 0.932, *η*_p_^2^ = 0.016] or training modality effects [F (1,28) = 0.457, *p* = 0.505, *η*_p_^2^ = 0.016] were found, however it was significantly affected by the factor time [F(3,26) = 55.115, *p* < 0.001, *η*_p_^2^ = 0.864].

Similarly, VAS ratings of muscle soreness (Figure 2h) showed no significant interaction effect [F(3,26) = 0.165, *p* = 0.189, *η*p^2^ = 0.165] and no significant main effect of training modality [F(1,28) = 0.882, *p* = 0.356, *η*p^2^ = 0.031], but a significant main effect of time [F(3,26) = 84.307, *p* < 0.001, *η*p^2^ = 0.907].

## Discussion

The present study investigated and compared the acute fatigue responses to two strength training sessions—traditional strength training (CR-BS) and a protocol emphasizing reactive plyometric training (PLY-BS)—in elite youth soccer players over a 72 h recovery period. Contrary to our hypothesis, no significant training modality × time interactions were observed for any of the measured outcome parameters, suggesting that both interventions elicited comparable fatigue trajectories across neuromuscular, biochemical, and subjective markers. Nonetheless, the significant time effects detected across multiple outcomes underscore the importance of monitoring recovery over several days. Even among youth athletes, who generally demonstrate faster recovery kinetics and higher adaptive potential compared to adults due to lower absolute mechanical strain and greater relative oxidative efficiency (68, 69), both training modalities induced measurable fatigue responses within the 72 h post-exercise period.

Consistent with previous findings (2, 22), both interventions induced marked impairments in neuromuscular performance within the first 24 h post-exercise, particularly evident in CMJ (−8.85%), IMTP (−17.29%), and DJ (−17.29%) performance. These decrements are indicative of fatigue affecting both contractile function and SSC efficiency. Although PLY-BS training is typically associated with shorter-lasting neuromuscular impairments due to lower mechanical and metabolic strain (25), our findings indicate that, in youth athletes with limited prior exposure to structured strength training, even moderate-volume plyometric loading can elicit marked neuromuscular fatigue lasting up to 48 h. This aligns with previous evidence showing that plyometric exercises, despite their dynamic and ballistic nature, can induce delayed fatigue responses when performed by unaccustomed individuals (70, 71).

CK levels increased markedly 24 h post-exercise, peaked at 48 h, and declined by 72 h—a pattern consistent with the delayed onset and resolution of muscle damage (21, 72). Although CK is a non-specific marker with high inter-individual variability (60), the observed time course in response to both protocols supports the notion that moderate mechanical tissue stress occurred following both training modalities.

Subjective fatigue markers, including Hooper Index and VAS, mirrored the objective performance decrements, peaking at 24 h and progressively recovering thereafter. This convergence shows the utility of subjective tools in monitoring training responses, especially in youth populations where invasive or equipment-heavy testing is not always feasible (52, 73, 74). Notably, participants reported similar levels of perceived exertion in both sessions, suggesting that internal load perception does not differ markedly between CR-BS and PLY-BS protocols when performed to volitional fatigue.

The AST and SR tests provided additional insight into fatigue responses in specific muscle regions. Hip adductor strength, as measured by the squeeze test, showed significant reductions post-exercise, with partial recovery by 72 h, corroborating recent findings on its sensitivity to hip and groin loading (47). SR scores exhibited only minor fluctuations, suggesting that posterior chain flexibility may be less sensitive to acute fatigue unless substantial muscle damage is present (46). Nonetheless, SR remains a valuable adjunct marker, particularly within holistic athlete monitoring schemes.

One potential explanation for the similar fatigue effects may lie in the limited prior experience of the players with structured strength training over longer period. As previously documented, training history is a critical moderator of both performance gains and fatigue responses, with less-experienced athletes often exhibiting heightened sensitivity to novel mechanical stimuli, irrespective of the specific modality used (10, 75). This is particularly relevant in youth populations, where neuromuscular coordination and load tolerance are still developing, potentially attenuating the specificity of adaptations (76). Consequently, the comparable fatigue responses observed in both CR-BS and PLY-BS conditions may reflect a generalized training stimulus for players with only a few experience in structured strength training, rather than modality-specific fatigue dynamics.

From a practical perspective, these findings underscore the importance of recovery planning in high-performance settings after strength training sessions. Both traditional and plyometric training programs can be safely integrated into youth soccer microcycles, provided that adequate recovery windows between strength training sessions are ensured. As most fatigue markers returned to near-baseline levels within 72 h, a recovery period of 2–3 days appears appropriate before subsequent high-intensity strength training sessions. In practice, this means that coaches can flexibly alternate traditional and plyometric-oriented sessions within the same microcycle, depending on the technical or tactical emphasis of the week, without increasing cumulative fatigue risk. At a macrocycle level, these findings support the continued use of structured strength training throughout the competitive season to maintain neuromuscular and physical performance, rather than restricting such training exclusively to preparatory phases (77, 78).

Several limitations of this study should be acknowledged and considered when interpreting the findings. The lack of long-term follow-up precludes conclusions regarding chronic adaptations. However, previous work by Kadlubowski et al. (2025) demonstrated that both training approaches—traditional strength training combined with calf raises and a program emphasizing reactive strength training—can produce comparable improvements in sprint performance, jump height, and maximal strength over a six-month period in youth soccer players. The present study complements those findings by showing that both protocols also induce similar short-term fatigue responses and may therefore be equally well integrated into weekly training schedules in elite youth soccer. The relatively high training volume in the present study, combined with the participants’ limited prior exposure to structured resistance training, likely contributed to the magnitude of neuromuscular and biochemical fatigue observed. Future studies should consider stratifying participants by strength training experience and systematically varying load and volume to better isolate the contribution of training background to fatigue and recovery dynamic. Furthermore, because both experimental sessions included the back squat, a substantial portion of the total workload was shared between conditions. While this design enhanced ecological validity by replicating actual soccer strength-training practice, it may have attenuated between-condition differences in some global fatigue markers. Another limitation is that CK and jump tests, while providing useful proxies for biochemical or neuromuscular status, do not fully capture central fatigue or sport-specific performance outcomes. In particular, the inclusion of linear sprint testing would have further strengthened the ecological validity of the monitoring battery, as sprint performance represents a key determinant of decisive match actions in soccer. However, sprint assessments were deliberately omitted to limit overall testing time and to avoid additional neuromuscular loading that might have interfered with the repeated follow-up measurements across the 72 h recovery period. Given the well-established associations between CMJ, DJ, IMTP variables and sprint ability in youth soccer players, the selected tests were considered appropriate surrogate markers of neuromuscular performance, although future studies should complement these with direct speed assessments. Finally, the absence of sleep and nutritional monitoring introduces potential confounding effects, as individual differences in recovery behavior may have influenced the observed fatigue responses.

Nonetheless, the study's crossover design, high measurement reliability, and the combined use of objective and subjective fatigue markers strengthens the study's internal validity. While improvements in sprint performance, jump height, and maximal strength following these training modalities have already been demonstrated (13), future studies should investigate how these physical adaptations translate to technical and tactical performance in youth soccer. Additionally, integrating central fatigue markers (e.g., heart rate variability) may help to monitor autonomic recovery status, which can be influenced by sleep and other recovery behaviors.

In conclusion, both CR-BS and PLY-BS protocols induced significant but transient fatigue across neuromuscular, biochemical, and subjective parameters in youth elite soccer players. No significant differences in fatigue responses were found between the strength training interventions. Therefore, both training protocols appear viable for integration into structured athletic development programs, provided that adequate recovery is allowed between sessions.

## Data Availability

The raw data supporting the conclusions of this article will be made available by the authors, without undue reservation.

## References

[1] Comfort P Stewart A Bloom L Clarkson B. Relationships between strength, sprint, and jump performance in well-trained youth soccer players. J Strength Cond Res. (2013) 28(1):173–177. 10.1519/JSC.0b013e318291b8c7 23542878

[2] Chatzinikolaou A Fatouros IG Gourgoulis V Avloniti A Jamurtas AZ Nikolaidis MG Time course of changes in performance and inflammatory responses after acute plyometric exercise. J Strength Cond Res. (2010) 24(5):1389–98. 10.1519/JSC.0b013e3181d1d318 20386477

[3] Faude O Koch T Meyer T. Straight sprinting is the most frequent action in goal situations in professional football. J Sports Sci. (2012) 30:625–31. 10.1080/02640414.2012.665940 22394328

[4] Haugen T Tønnessen E Hisdal J Seiler S. The role and development of sprinting speed in soccer. Int J Sports Physiol Perform. (2014) 9(3):432–41. 10.1123/ijspp.2013-0121 23982902

[5] de Hoyo M Gonzalo-Skok O Sanudo B Carrascal C Plaza-Armas J Camacho-Candil F Comparative effects of in-season full-back squat, resisted sprint training, and plyometric training on explosive performance in U-19 elite soccer players. J Strength Cond Res. (2016) 30(2):368–77. 10.1519/JSC.0000000000001094 26813630

[6] Keiner M Brauner T Kadlubowski B Sander A Wirth K. The influence of maximum squatting strength on jump and sprint performance: a cross-sectional analysis of 492 youth soccer players. Int J Environ Res Public Health (IJERPH). (2022) 19:5835. 10.3390/ijerph19105835 35627371 PMC9140541

[7] Otero-Esquina C de Hoyo Lora M Gonzalo-Skok Ó Domínguez-Cobo S Sánchez H. Is strength-training frequency a key factor to develop performance adaptations in young elite soccer players? Eur J Sport Sci. (2017) 17(10):1241–51. 10.1080/17461391.2017.1378372 28944722

[8] Crotty ED Furlong LM Harrison AJ. Ankle and plantar flexor muscle-tendon unit function in sprinters: a narrative review. Sports Med. (2024) 54(3):585–606. 10.1007/s40279-023-01967-1 37989833

[9] Pandy M Lai A Schache A Lin Y-C. How muscles maximize performance in accelerated sprinting. Scand J Med Sci Sports. (2021) 31:1–15. 10.1111/sms.14021 34270824

[10] Suchomel TJ Nimphius S Stone MH. The importance of muscular strength in athletic performance. Sports Med. (2016) 46(10):1419–49. 10.1007/s40279-016-0486-0 26838985

[11] Markovic G Mikulic P. Neuro-musculoskeletal and performance adaptations to lower-extremity plyometric training. Sports Med. (2010) 40(10):859–95. 10.2165/11318370-000000000-00000 20836583

[12] Ramirez-Campillo R Castillo D Raya-González J Moran J de Villarreal E S Lloyd R. Effects of plyometric jump training on jump and sprint performance in young male soccer players: a systematic review and meta-analysis. Sports Med. (2020) 50(12):2125–43. 10.1007/s40279-020-01337-1 32915430

[13] Kadlubowski B Keiner M Wirth K Csapo R. Effects of traditional strength vs. Combined strength and plyometric training on sprint, jump, and maximum strength performance in elite youth soccer players-a 6-month controlled trial. J Strength Cond Res. (2025) 39(7):878–89. 10.1519/jsc.0000000000005113 40440548

[14] Enoka RM Duchateau J. Muscle fatigue: what, why and how it influences muscle function. J Physiol. (2008) 586(1):11–23. 10.1113/jphysiol.2007.139477 17702815 PMC2375565

[15] Moran J Liew B Ramirez-Campillo R Granacher U Negra Y Chaabene H. The effects of plyometric jump training on lower-limb stiffness in healthy individuals: a meta-analytical comparison. J Sport Health Sci. (2023) 12(2):236–45. 10.1016/j.jshs.2021.05.005 34033984 PMC10105022

[16] Zając A Chalimoniuk M Maszczyk A Gołaś A Lngfort J. Central and peripheral fatigue during resistance exercise—a critical review. J Hum Kinet. (2015) 49:159–69. 10.1515/hukin-2015-0118 26839616 PMC4723165

[17] Kluger BM Krupp LB Enoka RM. Fatigue and fatigability in neurologic illnesses: proposal for a unified taxonomy. Neurology. (2013) 80(4):409–16. 10.1212/WNL.0b013e31827f07be 23339207 PMC3589241

[18] Barahona-Fuentes GDF Huerta Ojeda Á Jerez-Mayorga D. Effects of different methods of strength training on indicators of muscle fatigue during and after strength training: a systematic review. Motriz. (2020) 26(3):1–20. 10.1590/s1980-6574202000030063

[19] Gandevia SC. Spinal and supraspinal factors in human muscle fatigue. Physiol Rev. (2001) 81(4):1725–89. 10.1152/physrev.2001.81.4.1725 11581501

[20] Proske U Morgan DL. Muscle damage from eccentric exercise: mechanism, mechanical signs, adaptation and clinical applications. J Physiol. (2001) 537(Pt 2):333–45. 10.1111/j.1469-7793.2001.00333.x 11731568 PMC2278966

[21] Baird MF Graham SM Baker JS Bickerstaff GF. Creatine-kinase- and exercise-related muscle damage implications for muscle performance and recovery. J Nutr Metab. (2012) 2012:960363. 10.1155/2012/960363 22288008 PMC3263635

[22] Thomas K Brownstein CG Dent J Parker P Goodall S Howatson G. Neuromuscular fatigue and recovery after heavy resistance, jump, and sprint training. Med Sci Sports Exerc. (2018) 50(12):2526–35. 10.1249/mss.0000000000001733 30067591

[23] Robbins D Goodale T Docherty D Behm D Quân T. The effects of load and training pattern on acute neuromuscular responses in the upper body. J Strength Cond Res. (2010) 24:2996–3007. 10.1519/JSC.0b013e3181f67474 20975369

[24] Drinkwater EJ Lane T Cannon J. Effect of an acute bout of plyometric exercise on neuromuscular fatigue and recovery in recreational athletes. J Strength Cond Res. (2009) 23(4):1181–6. 10.1519/JSC.0b013e31819b79aa 19528848

[25] Krzysztofik M Wilk M Pisz A Kolinger D Bichowska M Zajac A Acute effects of high-load vs. plyometric conditioning activity on jumping performance and the muscle-tendon mechanical properties. J Strength Cond Res. (2023) 37(7):1397–403. 10.1519/jsc.0000000000004398 37347943

[26] Drayton AM Hamad MJ Spyrou K. The time course of postmatch physical impairments in professional soccer: a systematic review. J Strength Cond Res. (2025) 39(11):e1345–e55. 10.1519/jsc.0000000000005252 40845278

[27] Brauers JJ Den Hartigh RJR Klooster D Oosterveld FGJ Lemmink K Brink MS. The short-term relation between load and acute psychophysiological responses in football: a meta-analysis and methodological considerations. Sci Med Footb. (2025) 10(1):105–25. 10.1080/24733938.2025.2476474 40159621

[28] Nédélec M McCall A Carling C Legall F Berthoin S Dupont G. Recovery in soccer: part I—post-match fatigue and time course of recovery. Sports Med. (2012) 42(12):997–1015. 10.2165/11635270-000000000-00000 23046224

[29] Brownstein CG Dent JP Parker P Hicks KM Howatson G Goodall S Etiology and recovery of neuromuscular fatigue following competitive soccer match-play. Front Physiol. (2017) 8:831. 10.3389/fphys.2017.00831 29118716 PMC5661001

[30] Foster C Florhaug JA Franklin J Gottschall L Hrovatin LA Parker S A new approach to monitoring exercise training. J Strength Cond Res. (2001) 15(1):109–15. 10.1519/00124278-200102000-00019 11708692

[31] Impellizzeri FM Rampinini E Coutts AJ Sassi A Marcora SM. Use of RPE-based training load in soccer. Med Sci Sports Exerc. (2004) 36(6):1042–7. 10.1249/01.Mss.0000128199.23901.2f 15179175

[32] Baker J Wilson S Johnston K Dehghansai N Koenigsberg A de Vegt S Talent research in sport 1990–2018: a scoping review. Front Psychol. (2020) 11:607710. 10.3389/fpsyg.2020.607710 33324305 PMC7723867

[33] Swann C Moran A Piggott D. Defining elite athletes: issues in the study of expert performance in sport psychology. Psychol Sport Exerc. (2015) 16:3–14. 10.1016/j.psychsport.2014.07.004

[34] Brodt V Wagner DR Heath EM. Countermovement vertical jump with drop step is higher than without in collegiate football players. J Strength Cond Res. (2008) 22(4):1382–5. 10.1519/JSC.0b013e3181739496 18545162

[35] Barker LA Harry JR Mercer JA. Relationships between countermovement jump ground reaction forces and jump height, reactive strength Index, and jump time. J Strength Cond Res. (2018) 32(1):248–54. 10.1519/jsc.0000000000002160 28746248

[36] Turner A Walker S Stembridge M Coneyworth P Reed G Birdsey L A testing battery for the assessment of fitness in soccer players. Strength Cond J. (2011) 33(5):29–39. 10.1519/SSC.0b013e31822fc80a

[37] Weakley J Black G McLaren S Scantlebury S Suchomel T McMahon E Testing and profiling athletes: recommendations for test selection, implementation, and maximizing information. Strength Cond J. (2023) 46(2):159–79. 10.1519/SSC.0000000000000784

[38] Springham M Singh N Stewart P Matthews J Jones I Norton-Sherwood C Greater efficacy for countermovement jump height than flight time: contraction ratio measures for signalling match-induced neuromuscular fatigue in english premier league academy football players. European College of Sport Science Annual Conference. Glasgow, Scotland (2024).

[39] Heishman A Brown B Daub B Miller R Freitas E Bemben M. The influence of countermovement jump protocol on reactive strength Index modified and flight time: contraction time in collegiate basketball players. Sports (Basel). (2019) 7(2):37. 10.3390/sports7020037 30759731 PMC6410267

[40] Lonergan B Cohen D Williams S Lawson R Howarth D Johnson D. Inter-day reliability of countermovement jump metrics in elite academy soccer players. Int J Strength Cond. (2025) 5:1–11. 10.47206/ijsc.v5i1.504

[41] Keiner M Sander A Wirth K Hartmann H. Differences in the performance tests of the fast and slow stretch and shortening cycle among professional, amateur and elite youth soccer players. J Hum Sport Exerc. (2015) 10:563–70. 10.14198/jhse.2015.102.03

[42] Quagliarella L Sasanelli N Belgiovine G Accettura D Notarnicola A Moretti B. Evaluation of counter movement jump parameters in young male soccer players. J Appl Biomater Biomech. (2011) 9(1):40–6. 10.5301/jabb.2011.7732 21607936

[43] Sierra-Casas A Rodríguez-Marroyo JA Castillo D Gutiérrez-Arroyo J Rodríguez-Fernández A. From load monitoring to training decisions: a practical approach using drop jump metrics in semi-professional soccer. BMC Sports Sci Med Rehabil. (2025) 17(1):301. 10.1186/s13102-025-01356-3 41107876 PMC12532431

[44] Konrad A Kasahara K Yoshida R Yahata K Sato S Murakami Y Relationship between eccentric-exercise-induced loss in muscle function to muscle soreness and tissue hardness. Healthcare. (2022) 10(1):96. 10.3390/healthcare10010096 35052259 PMC8775922

[45] Donti O Konrad A Panidi I Dinas PC Bogdanis GC. Is there a “window of opportunity” for flexibility development in youth? A systematic review with meta-analysis. Sports Med Open. (2022) 8(1):88. 10.1186/s40798-022-00476-1 35792993 PMC9259532

[46] Cuenca-Garcia M Marin-Jimenez N Perez-Bey A Sánchez-Oliva D Camiletti-Moiron D Alvarez-Gallardo IC Reliability of field-based fitness tests in adults: a systematic review. Sports Med. (2022) 52(8):1961–79. 10.1007/s40279-021-01635-2 35064915

[47] McMinn K Diewald S Harrison C Cronin J Ye-Lee D. Inter- and intra-session variability of compression strain gauge for the adductor groin squeeze test on soccer athletes. Healthc Technol Lett. (2024) 11(1):16–20. 10.1049/htl2.12074 38370163 PMC10869877

[48] Jiang D Liu Z Ling X Dai J Long L Lu Y Investigating the impact of inter-limb asymmetry in hamstring strength on jump, sprint, and strength performance in young athletes: comparing the role of gross force. Front Physiol. (2023) 14:1185397. 10.3389/fphys.2023.1185397 37304819 PMC10248013

[49] Hooper SL Mackinnon LT. Monitoring overtraining in athletes. Sports Med. (1995) 20(5):321–7. 10.2165/00007256-199520050-00003 8571005

[50] Rabbani A Clemente FM Kargarfard M Chamari K. Match fatigue time-course assessment over four days: usefulness of the hooper index and heart rate variability in professional soccer players. Front Physiol. (2019) 10:109. 10.3389/fphys.2019.00109 30837890 PMC6390199

[51] Clemente FM Mendes B Nikolaidis PT Calvete F Carriço S Owen AL. Internal training load and its longitudinal relationship with seasonal player wellness in elite professional soccer. Physiol Behav. (2017) 179:262–7. 10.1016/j.physbeh.2017.06.021 28668619

[52] Haddad M Stylianides G Djaoui L Dellal A Chamari K. Session-RPE method for training load monitoring: validity, ecological usefulness, and influencing factors. Front Neurosci. (2017) 11:612. 10.3389/fnins.2017.00612 29163016 PMC5673663

[53] Arcos AL Yanci J Mendiguchia J Gorostiaga EM. Rating of muscular and respiratory perceived exertion in professional soccer players. J Strength Cond Res. (2014) 28(11):3280–8. 10.1519/jsc.0000000000000540 24845209

[54] Gomez-Piriz PT Jiménez-Reyes P Ruiz-Ruiz C. Relation between total body load and session rating of perceived exertion in professional soccer players. J Strength Cond Res. (2011) 25(8):2100–3. 10.1519/JSC.0b013e3181fb4587 21685808

[55] Rodríguez-Marroyo JA Antoñan C. Validity of the session rating of perceived exertion for monitoring exercise demands in youth soccer players. Int J Sports Physiol Perform. (2015) 10(3):404–7. 10.1123/ijspp.2014-0058 25202917

[56] Crawford DA Drake NB Carper MJ DeBlauw J Heinrich KM. Validity, reliability, and application of the session-RPE method for quantifying training loads during high intensity functional training. Sports (Basel). (2018) 6(3):84. 10.3390/sports6030084 30134535 PMC6162783

[57] Li Z Qin S Li X Ren D. Use the rating of perceived exertion to evaluate load in collegiate male soccer player, validity and influencing factors. Sci Rep. (2025) 15(1):16880. 10.1038/s41598-025-01942-y 40374811 PMC12081865

[58] Lee KA Hicks G Nino-Murcia G. Validity and reliability of a scale to assess fatigue. Psychiatry Res. (1991) 36(3):291–8. 10.1016/0165-1781(91)90027-m 2062970

[59] Delgado DA Lambert BS Boutris N McCulloch PC Robbins AB Moreno MR Validation of digital visual analog scale pain scoring with a traditional paper-based visual analog scale in adults. J Am Acad Orthop Surg Glob Res Rev. (2018) 2(3):e088. 10.5435/JAAOSGlobal-D-17-00088 30211382 PMC6132313

[60] Brancaccio P Lippi G Maffulli N. Biochemical markers of muscular damage. Clin Chem Lab Med. (2010) 48(6):757–67. 10.1515/cclm.2010.179 20518645

[61] Ra SG Miyazaki T Kojima R Komine S Ishikura K Kawanaka K Effect of BCAA supplement timing on exercise-induced muscle soreness and damage: a pilot placebo-controlled double-blind study. J Sports Med Phys Fitness. (2018) 58(11):1582–91. 10.23736/s0022-4707.17.07638-1 28944645

[62] Hagstrom AD Shorter KA. Creatine kinase, neuromuscular fatigue, and the contact codes of football: a systematic review and meta-analysis of pre- and post-match differences. Eur J Sport Sci. (2018) 18(9):1234–44. 10.1080/17461391.2018.1480661 29870313

[63] Haller N Blumkaitis JC Strepp T Schmuttermair A Aglas L Simon P Comprehensive training load monitoring with biomarkers, performance testing, local positioning data, and questionnaires—first results from elite youth soccer. Front Physiol. (2022) 13:1000898. 10.3389/fphys.2022.1000898 36262260 PMC9573975

[64] Mendes B Clemente FM Calvete F Carriço S Owen A. Seasonal training load monitoring among elite level soccer players: perceived exertion and creatine kinase variations between microcycles. J Hum Kinet. (2022) 81:85–95. 10.2478/hukin-2022-0008 35291628 PMC8884884

[65] Schoenfeld BJ Grgic J Van Every DW Plotkin DL. Loading recommendations for muscle strength, hypertrophy, and local endurance: a re-examination of the repetition Continuum. Sports (Basel). (2021) 9(2):32. 10.3390/sports9020032 33671664 PMC7927075

[66] Myer G Ford K McLean S Hewett T. The effects of plyometric vs dynamic stabilization and balance training on lower extremity biomechanics. Am J Sports Med. (2006) 34:445–55. 10.1177/0363546505281241 16282579

[67] Cohen J. Statistical Power Analysis for the Behavioral Sciences. New York: Lawrence Erlbaum Associates (1988). 10.4324/9780203771587

[68] Falk B Dotan R. Child-adult differences in the recovery from high-intensity exercise. Exerc Sport Sci Rev. (2006) 34(3):107–12. 10.1249/00003677-200607000-00004 16829737

[69] Ratel S Duché P Williams CA. Muscle fatigue during high-intensity exercise in children. Sports Med. (2006) 36(12):1031–65. 10.2165/00007256-200636120-00004 17123327

[70] Silva JR Rumpf MC Hertzog M Castagna C Farooq A Girard O Acute and residual soccer match-related fatigue: a systematic review and meta-analysis. Sports Med. (2018) 48(3):539–83. 10.1007/s40279-017-0798-8 29098658

[71] Twist C Eston R. The effects of exercise-induced muscle damage on maximal intensity intermittent exercise performance. Eur J Appl Physiol. (2005) 94:652–8. 10.1007/s00421-005-1357-9 15887020

[72] Deli CK Fatouros IG Paschalis V Georgakouli K Zalavras A Avloniti A A comparison of exercise-induced muscle damage following maximal eccentric contractions in men and boys. Pediatr Exerc Sci. (2017) 29(3):316–25. 10.1123/pes.2016-0185 28165870

[73] Kellmann M Bertollo M Bosquet L Brink M Coutts AJ Duffield R Recovery and performance in sport: consensus statement. Int J Sports Physiol Perform. (2018) 13(2):240–5. 10.1123/ijspp.2017-0759 29345524

[74] Saw AE Main LC Gastin PB. Monitoring the athlete training response: subjective self-reported measures trump commonly used objective measures: a systematic review. Br J Sports Med. (2016) 50(5):281–91. 10.1136/bjsports-2015-094758 26423706 PMC4789708

[75] Faigenbaum AD Kraemer WJ Blimkie CJ Jeffreys I Micheli LJ Nitka M Youth resistance training: updated position statement paper from the national strength and conditioning association. J Strength Cond Res. (2009) 23(5 Suppl):S60–79. 10.1519/JSC.0b013e31819df407 19620931

[76] Lloyd RS Oliver JL Faigenbaum AD Howard R De Ste Croix MB Williams CA Long-term athletic development- part 1: a pathway for all youth. J Strength Cond Res. (2015) 29(5):1439–50. 10.1519/jsc.0000000000000756 25486295

[77] Rønnestad BR Nymark BS Raastad T. Effects of in-season strength maintenance training frequency in professional soccer players. J Strength Cond Res. (2011) 25(10):2653–60. 10.1519/JSC.0b013e31822dcd96 21873897

[78] Wing C. In-Season strength and power training considerations for professional soccer teams competing within national level competitions. Strength Cond J. (2018) 40(3):12–22. 10.1519/ssc.0000000000000377

