# Supplementary material for: Acute effects of strength training interventions on subjective, neuromuscular, and biochemical fatigue parameters in elite youth soccer players
Source: Front Sports Act Living. 2026 Mar 4;8:1742295. doi: 10.3389/fspor.2026.1742295 (PMC12996250; doi:10.3389/fspor.2026.1742295)
Supplement: Supplementary file 1 [file Table1.docx]

| Training interventions for the U19 and U17 teams (Schedule) |
| --- |

| **14.04**  **Monday** | **15.04**  **Tuesday** | **16.04**  **Wednesday** | **17.04**  **Thursday** | **18.04**  **Friday** | **19.04**  **Saturday** | **20.04**  **Sunday** |
| --- | --- | --- | --- | --- | --- | --- |
| Pre-testing  Training intervention (Day 1)  Session RPE  (<30 Minutes after training) | Post-testing  (24 hours)  Video analysis | Post-testing  (48 hours)  Video analysis | Post-testing  (72 hours)  Soccer Training after testing  1. Coordinative warm up (10 minutes)  2. Passing exercise over short and long distance up to 30m (10 minutes)  3. Soccer tennis tournament (30 minutes) | Soccer Training  1. Activation in the gym (Mobilisation and stretching (20 minutes))  2. Technical exercises (10 minutes)  3. Tactical game in one half (3 times 5 minutes with 2 minutes break between)  4. Shooting exercise (10 minutes) | Soccer Training  1. Coordinative warm up at the ladder (10 minutes)  2. Technical parcour with different coordinative requirements (15 minutes)  3. Rondo (10 Minutes)  4. Positional exercise (20 Minutes) | **OFF** |
| **21.04** | **22.04** | **23.04** | **24.04** | **25.04** | **26.04** | **27.04** |
| **Off** | Pre-testing  Training intervention (Day 2)  Session RPE  (<30 Minutes after training) | Post-testing  (24 hours)  Video analysis | Post-testing  (48 hours)  Video analysis | Post-testing  (72 hours)  Soccer Training after testing |  |  |

*RPE = Session rating of perceived exertion; Off = no training session.*
